# Differential Expression of ADP/ATP Carriers as a Biomarker of Metabolic Remodeling and Survival in Kidney Cancers

**DOI:** 10.3390/biom11010038

**Published:** 2020-12-30

**Authors:** Lucia Trisolini, Luna Laera, Maria Favia, Antonella Muscella, Alessandra Castegna, Vito Pesce, Lorenzo Guerra, Anna De Grassi, Mariateresa Volpicella, Ciro Leonardo Pierri

**Affiliations:** 1 Department of Biosciences, Biotechnologies, Biopharmaceutics, University “Aldo Moro” of Bari, Via E. Orabona, 4, 70125 Bari, Italy; lucia.trisolini@uniba.it (L.T.); luna.laera@uniba.it (L.L.); mariafavia@hotmail.com (M.F.); alessandra.castegna@uniba.it (A.C.); vito.pesce@uniba.it (V.P.); lorenzo.guerra1@uniba.it (L.G.); 2 Dipartimento di Scienze e Tecnologie Biologiche e Ambientali (Di.S.Te.B.A.), Università del Salento, 73100 Lecce, Italy; antonella.muscella@unisalento.it; 3 BROWSer S.r.l. c/o, Department of Biosciences, Biotechnologies, Biopharmaceutics, University “Aldo Moro” of Bari, Via E. Orabona, 4, 70126 Bari, Italy

**Keywords:** ADP/ATP carriers (AACs), SLC25A family, mitochondrial apoptosis, mitochondrial permeability transition pore (mPTP), kidney cancer, biomarkers of survival, biomarkers of metabolic remodeling

## Abstract

ADP/ATP carriers (AACs) are mitochondrial transport proteins playing a strategic role in maintaining the respiratory chain activity, fueling the cell with ATP, and also regulating mitochondrial apoptosis. To understand if AACs might represent a new molecular target for cancer treatment, we evaluated AAC expression levels in cancer/normal tissue pairs available on the Tissue Cancer Genome Atlas database (TCGA), observing that AACs are dysregulated in most of the available samples. It was observed that at least two AACs showed a significant differential expression in all the available kidney cancer/normal tissue pairs. Thus, we investigated AAC expression in the corresponding kidney non-cancer (HK2)/cancer (RCC-Shaw and CaKi-1) cell lines, grown in complete medium or serum starvation, for investigating how metabolic alteration induced by different growth conditions might influence AAC expression and resistance to mitochondrial apoptosis initiators, such as “staurosporine” or the AAC highly selective inhibitor “carboxyatractyloside”. Our analyses showed that AAC2 and AAC3 transcripts are more expressed than AAC1 in all the investigated kidney cell lines grown in complete medium, whereas serum starvation causes an increase of at least two AAC transcripts in kidney cancer cell lines compared to non-cancer cells. However, the total AAC protein content is decreased in the investigated cancer cell lines, above all in the serum-free medium. The observed decrease in AAC protein content might be responsible for the decrease of OXPHOS activity and for the observed lowered sensitivity to mitochondrial apoptosis induced by staurosporine or carboxyatractyloside. Notably, the cumulative probability of the survival of kidney cancer patients seriously decreases with the decrease of AAC1 expression in KIRC and KIRP tissues making AAC1 a possible new biomarker of metabolic remodeling and survival in kidney cancers.

## 1. Introduction

Most current cancer chemotherapeutics cause cell death via apoptosis [1]. Two main apoptotic pathways have been described in great detail, the extrinsic or death receptor pathway, and the intrinsic or mitochondrial pathway [2]. It is known that mitochondrial apoptosis is triggered by the opening of mitochondrial permeability transition pore complex (mPTP) and ADP/ATP mitochondrial carriers (AACs, [3,4]) play a key role in regulating mPTP opening and thus in triggering the mitochondrial apoptotic pathway [3,4,5,6,7].

Four genes coding for AACs, namelyAAC1 encoded by *SLC25A4*; AAC2 encoded by *SLC25A5*; AAC3 encoded by *SLC25A6*; and AAC4 encoded by *SLC25A31*) have been found in *Homo sapiens*. Each AAC gene displays a tissue-specific expression patternas follows: AAC1 is highly expressed in skeletal muscles, heart, and brain; AAC2 is specifically expressed in undifferentiated cells, such as lymphocytes, or in tissues that are able to proliferate and regenerate, such as kidney and liver; AAC3 is ubiquitously expressed in all tissues; AAC4 is a germ-cell isoform [4,8]. Tissue-specific AAC expression patterns are similar in different mammals and may be related to tissue-specific energy requirements [8].

Notably, AAC1 was the first member of the Mitochondrial Carrier Family (MCF [9,10]) to be isolated, cloned, and reconstituted into liposomes [4,9,11]. Furthermore, the bovine AAC1 paralog was crystallized in its cytosolic conformation in complex with its cytosolic-face specific powerful inhibitor carboxyatractyloside (CXT, also known as CATR, [3,4,12,13,14]). Recently, the AAC1 from *Thermothelomyces thermophilus* was successfully crystallized in its matrix conformation in complex with its matrix-face specific inhibitor bongkrekic acid (BKA, [3,4,13,14,15]).

The full-length sequences of the four human AAC genes share between 89 and 95% of identical amino acids with the crystallized bovine AAC full-length sequence. Given the high percentage of identical amino acids with the crystallized bovine AAC1, it was possible to characterize the binding region of the human AACs [4,16] and estimate in vitro the affinity of CXT (4 nM, competitive inhibitor) and BKA (2 µM, non-competitive inhibitor) for the human AAC2. Furthermore, it was possible to predict in silico new AAC selective inhibitors, namely steviol (Ki = 25 µM, competitive inhibitor, already known for its ability in inhibiting ATP synthesis in mitochondria [17]), suramine (Ki = 0.2 µM, competitive inhibitor), and chebulinic acid (Ki = 2 µM, competitive inhibitor) whose affinities for the human AAC2 were determined through in vitro transport assays on the recombinant human AAC2 reconstituted in proteoliposomes [4,16].

The availability of such molecules that trigger apoptosis by acting as high affinity inhibitors of the human AAC2 might represent a novel apoptosis control tool to be used in diseases showing an altered apoptosis process, like cancer. Notably, it was observed that CXT efficiently induces mitochondrial dysfunction at the molecular level and poisoning at the physiological level [18,19,20].

Thus, AAC2’s role as an anti-cancer target was investigated in a preclinical mouse model of a colon adenocarcinoma xenograft showing AAC2 pharmacological/genetic targeting improved the results of traditional chemotherapy [21,22,23].

Considering the cited data with specific reference to the impaired expression of AACs in several cancers [24,25] and the natural trend of CXT to accumulate at kidney level and cause multiple organ dysfunction [18], we asked wether CXT, a highly selective AAC inhibitor, could represent a pharmacological option for those cancers showing dysregulated AACs and mitochondrial impairment. With this aim, we screened the The Cancer Genome Atlas database (TCGA) for quantifying AAC expression levels in the TCGA cancer/normal tissue pairs. The screening revealed that AACs are dysregulated in several cancer tissues. Notably, all the available kidney cancer/normal tissue pairs showed dysregulated AACs. Thus, we decided to investigate AAC expression in kidney cells by quantifying the expression levels of AACs in one kidney non-cancer (HK2) and two cancer (RCC-Shaw and CaKi-1) kidney cell lines grown in complete medium or serum starvation.

Furthermore, we quantified the ability of CXT in inducing AAC-mediated mitochondrial apoptosis in the investigated kidney cell lines.

## 2. Materials and Methods

### 2.1. Cell Culture

Human cortex/proximal tubule kidney cells (HK2) and Human Renal Cancer cells (CaKi-1) [26] were obtained from the American Type Culture Collection. Renal carcinoma cells (RCC-Shaw) are primary RCC cell lines established from primary kidney tissue explants derived from biopsy and currently provided by Public Health England (PHE)—culture collections [27].

HK2 and CaKi-1 cells were maintained in high glucose Dulbecco’s Modified Eagle’s Medium (DMEM) with sodium pyruvate and stable glutamine (Euroclone, ECL0103L, Pero, Milan, Italy), supplemented with 10% Fetal Bovine Serum (FBS, Euroclone ECS0180L, Pero, Milan, Italy), and 1% penicillin-streptomycin (Euroclone, ECM0010, Pero, Milan, Italy).

RCC-Shaw cells were grown in Roswell Park Memorial Institute medium (RPMI 1640) with a stable glutamine (Euroclone, ECM2001L, Pero, Milan, Italy) supplement with 10% FBS (Euroclone ECS0180L, Pero, Milan, Italy) and 1% penicillin-streptomycin (Euroclone, ECM0010, Pero, Milan, Italy). All cells were cultured in a humidified atmosphere with 5% CO_2_ at 37 °C.

All the cited cell lines were also grown in starved conditions. For obtaining the starvation, the growth medium was removed, and the cells were washed twice with serum-free or Phosphate-buffered saline (PBS-Lonza, LONZ17-517Q, Lonza Group Ltd. Basel Switzerland). Then, the cells were maintained for 24 h in the above-cited DMEM medium without FBS according to [28].

### 2.2. RNA Quantification in Tissues from TCGA Database

RNA expression levels of genes coding for AACs (*SLC25A4*, *SLC25A5*, *SLC25A6*, and *SLC25AA31*) in normal and cancer tissues were obtained from the TCGA database and analyzed through Timer2 [29] (http://timer.cistrome.org/->CANCER EXPLORATION->Gene_DE). A total of 21 pairs of normal/cancer tissues were analyzed for estimating the expression of the investigated genes. Notably, cancer/normal tissue sample pairs were collected starting from tumor and adjacent normal tissues sampled from over 11,000 patients in 12 years [29,30]. Furthermore, Timer2 [29] was also used for estimating the correlation between the expression levels of the investigated AACs and the survival rate of patients affected by kidney cancer (as observed from patients whose samples are available on the TCGA database). More in detail, cumulative survival probability in patients with the highest or lowest 50% *SLC25A4*, *SLC25A5*, and *SLC25A6* expression was evaluated using Kaplan–Meier curves and log-rank comparison (http://timer.cistrome.org/->CANCER EXPLORATION->Gene_Outcome).

### 2.3. RNA Extraction and qRT-PCR of the Investigated Kidney Cell Lines Grown in Complete Medium or Starved (Serum-Free) Conditions

Total RNA was extracted using the AURUM Total RNA Mini Kit (732-6820 Biorad, Hercules, CA, USA). First-strand cDNA synthesis was synthesized starting from 1 μg of RNA and using the iScript Reverse Transcription Supermix for RT-qPCR kit (1708840, Biorad, Hercules, CA, USA), according to manual instructions. qRT-PCR experiments were performed on the QuantStudio6Flex Real-Time PCR System (AppliedBiosystems, LifeTechnologies, Waltham, MA, USA), using 1 µL of diluted cDNA (1:3) as the template for each reaction with SYBR Green PCR Master Mix (LifeTechnologies, Carlsbad, CA, USA). PCR amplifications were performed using the pairs of primers reported in Supplementary Table S1. Gene coding for actin (ACT) was used as a reference gene [31] (Supplementary Table S1). Data from qRT-PCR experiments for both the endogenous and control genes are the mean values of three independent amplification reactions carried out on two different biological replicates harvested at the same cellular growth (according to AURUM kit protocols). The specificity of the amplicons was confirmed by the presence of a single band of expected size for each primer pair in agarose gels (2% *w*/*v*), by single peak melting curves of the PCR products, and by the sequencing of the amplified fragments (Macrogen, Amsterdam, The Netherlands). Fluorescence raw data were exported by the Flex Real Time PCR System Software (Applied Biosystems, LifeTechnologies, Waltham, MA, USA) and analyzed as already reported in [32].

### 2.4. Protein Extraction

Cells from the investigated cell lines (HK2, CaKi-1, and RCC-Shaw) grown in T75 flasks were rinsed in PBS, trypsinized, and collected in centrifuge tubes. Then the collected cells were centrifuged at 9000 rpm for 5 min. The supernatant was eliminated, and the pellet was resuspended in 100 μL of RIPA buffer (50 μL for 1 million cells—Burker chamber counted) that consisted of 50 mM Tris HCl (pH = 8), 150 mM Sodium Chloride, 1.0% NP 40, 0.5% sodium deoxychloride, 0.1% sodium dodecyl sulfate.

About 40 passages with a sterile syringe were performed to break the cells and then the obtained mix was sonicated for about 6 s. The obtained mix, containing broken cells, was centrifuged for 15 min at 12,000 rpm and the concentration was evaluated with the Bradford method.

### 2.5. Immunoblotting Assays

In total, 20 μL of cell lysates coming from the resuspension with the RIPA buffer were mixed with Laemmli buffer (161-074, Biorad, Hercules, CA, USA) according to the datasheet and denatured at 95 °C for 5 min [33]. After SDS-PAGE (TGX Stain-Free FastCast Acrylamide Kit, 12% #1610185—Biorad, Hercules, CA, USA), proteins were transferred onto a nitrocellulose membrane (88018, Thermo Fisher Scientific, Waltham, MA, USA) using the Criterion Blotter (Bio-Rad Laboratories, Hercules, CA, USA). After the protein transfer, membranes were incubated with a blocking solution (5% milk-TBS, Hercules, CA, USA) and probed overnight, under shaking, at 4° C with anti-AAC2/SLC25A5 (Cell Signaling; #14671; 1:10,000 dilution, Danver, MA, USA) and anti-COXII (Invitrogen; #A-6404; 1:10,000 dilution, Carlsbad, CA, USA) primary antibodies. It should be noted that just one antibody was used for quantifying AAC proteins because all the available AAC antibodies are not able to recognize selectively a single paralog, due to the high percentage of identical residues (more than 94% of identical residues) among the investigated AACs. The following day, membranes were washed (0.5% Tween20-TBS) under shaking and incubated for 1 h at room temperature with appropriate peroxidase-conjugated secondary antibodies (Santa Cruz Biotechnology, Santa Cruz, CA, USA, 1:20,000 dilution). A mild stripping protocol (Stripping for reprobing, Abcam protocols) was used before incubating the membrane with a primary anti-actin antibody (A2066, Sigma Aldrich, Milan, Italy, 1:20,000 dilution) overnight. The following day, membranes were washed in 0.5% Tween20-TBS (P9416 and T5912, Sigma, St. Louis, MO, USA) under shaking and incubated for 1 h at room temperature with appropriate peroxidase-conjugated secondary antibodies (Santa Cruz Biotechnology, Santa Cruz, CA, USA; 1:30,000 dilution). Blots were visualized using the ECL Plus Western Blotting Detection Reagents and ECL films (GE Healthcare, Chicago, IL, USA). Autoradiographs were acquired by the ChemiDoc MP Imaging System and analyzed by Quantity One software (Bio-Rad Laboratories, Hercules, CA, USA). The densitometric value of optical density (OD) units of each protein band immunodetected was then related to the corresponding actin signal intensity (loading control) or mitochondrial COXII signal intensity and normalized by comparison to serum deprivation (starved conditions).

### 2.6. Caspase-9 Analysis

The three investigated cell lines (HK2, CaKi-1, and RCC-Shaw) grown on 60 mm dishes were treated with the powerful intrinsic apoptosis (cell death) initiator “staurosporine” (2 µM, 3 h incubation) [34] or with the AAC high selective inhibitor “CXT” (40 µM, 24 h incubation) [4,18]. The incubation time at which it was possible to observe the 25% of detached cells was used as a reference time threshold for apoptosis triggering [35,36].

After the indicated incubation times and supernatant removal, the cells were trypsinized and lysed in RIPA Buffer (as above described). 30 µg of proteins of each sample were separated on 10% stain-free polyacrylamide gels (Bio-Rad Laboratories, Inc., Hercules, CA, USA) under reducing conditions. Protein bands were electrophoretically transferred onto Immobilon-P membranes (Merck KGaA, Darmstadt, Germany) for Western blot analysis, blocked in TBS-Tween-20 containing 3% bovine serum albumin (BSA) and incubated with primary antibodies against the cleaved human Caspase-9 p35 9 (D315) diluted 1:1000 (https://www.antibodies.com/it/cleaved-caspase-9-p35-d315-antibody-a34334), for the detection of mitochondrial apoptosis [37]. Immunoreactive bands were detected with secondary goat anti-mouse horseradish peroxidase–coupled antibodies. Membranes were incubated with Clarity Max ECL Western Blotting Substrates (Bio-Rad Laboratories, Inc., Hercules, CA, USA), and the signals were visualized with the ChemiDoc System gels (Bio-Rad Laboratories, Inc., Hercules, CA, USA). Obtained bands were normalized to total protein using stain-free technology gels (Bio-Rad Laboratories, Inc., Hercules, CA, USA).

## 3. Results

### 3.1. Differential Expression of AACs in Cancer/Normal Tissues from TCGA Analyses

**Figure 1:**
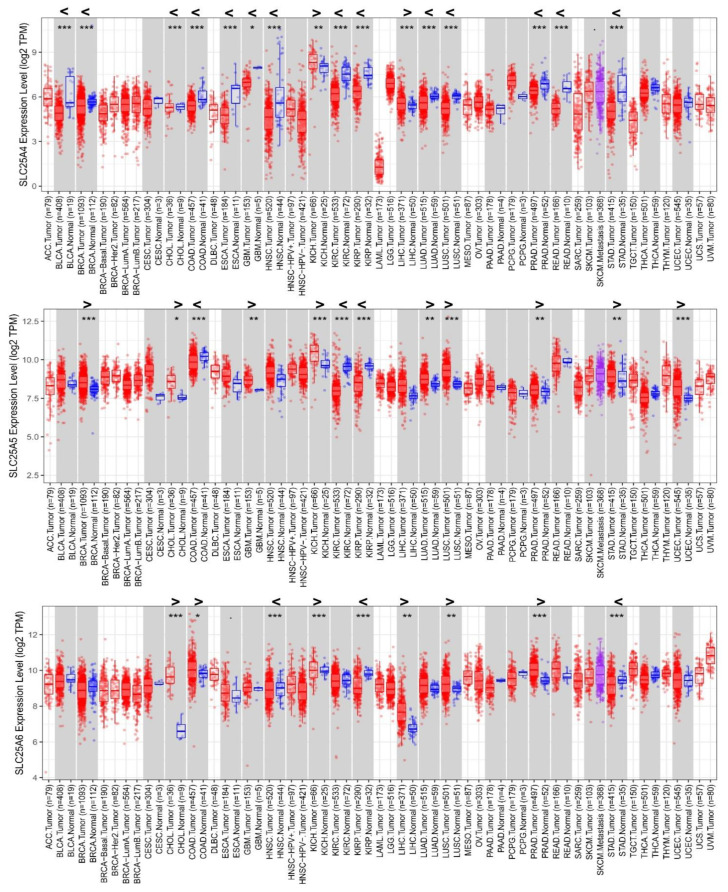
*SLC25A4_AAC1*, *SLC25A5_AAC2*, *SLC25A6_AAC3* expression levels in 21 “cancer vs. normal” tissue pairs available on TCGA, analyzed through Timer2. The 21 “cancer (red box plots) vs. normal (blue box plots)” tissue pairs are reported and displayed on a grey background. Cancer tissues without a normal counterpart are reported and displayed on a white background. The violet box plot indicates AAC expression levels in the metastatic SKCM tissue within the “tumor/metastasis” SKCM tissue pair. For a list of the abbreviations, please see https://gdc.cancer.gov/resources-tcga-users/tcga-code-tables/tcga-study-abbreviations. The Gene_Differential Expression (DE) module allows users to study the differential expression between tumor and adjacent normal tissues for any gene of interest across all TCGA tumors. Distributions of AAC gene expression levels are displayed using box plots. The statistical significance computed by differential analysis (edgeR) on RNA-Seq raw counts is annotated by the number of stars (*: *p*-value < 0.05; **: *p*-value < 0.01; ***: *p*-value < 0.001). Readers can identify upregulated (>) or downregulated (<) AAC genes in the tumors compared to normal tissues for each cancer type, as displayed in gray highlighted columns when normal data are available. The “n” values reported below the boxplots refer to the number of patients whose cancer/normal tissue sample pairs are available in the TCGA database.

Timer2 analysis shows that *SLC25A4*, *SLC25A5*, and *SLC25A6* genes are differentially expressed in 16, 12, and 9 tissues out of the available 21 “tumor vs. normal” tissue pairs, respectively (Figure 1). *SLC25A4* results significantly upregulated in 2 cancer tissues and significantly downregulated in 14 cancer tissues, out of the 21 “tumor vs. normal” tissue pairs (Figure 1, upper panel). *SLC25A5* results significantly upregulated in 9 cancer tissues and significantly downregulated in 3 cancer tissues, out of the 21 “tumor vs. normal” tissue pairs (Figure 1, middle panel). *SLC25A6* results significantly upregulated in 7 cancer tissues and significantly downregulated in 2 cancer tissues, out of the 21 “tumor vs. normal” tissue pairs (Figure 1, bottom panel). *SLC25A31* results poorly expressed in the 21 “tumor vs. normal” tissue pairs (Supplementary Figure S1).

More in detail, AAC1 is significantly (*p* < 0.001) downregulated in bladder urothelial carcinoma (BLCA), breast invasive carcinoma (BRCA), colon adenocarcinoma (COAD), kidney renal or renal papillary cell carcinoma (KIRC, KIRP), cholangiocarcinoma (CHOL), esophageal carcinoma (ESCA), head and neck squamous cell carcinoma (HNSC), lung adenocarcinoma or squamous cell carcinoma (LUAD, LUSC), prostate adenocarcinoma (PRAD), rectum adenocarcinoma (READ), and stomach adenocarcinoma (STAD) as observed in the available TCGA cancer tissues compared to the corresponding non-tumor counterpart (Figure 1).

Conversely, AAC2 is significantly upregulated in BRCA, kidney chromophobe cancer (KICH), LUSC, and uterine corpus endometrial carcinoma (UCEC), at variance with AAC3 significantly upregulated in CHOL, KICH, and PRAD (Figure 1).

Notably, AAC2 is significantly downregulated in COAD, KIRC, KIRP, whereas AAC3 is significantly downregulated in HNSC, KIRP, and STAD, respectively (Figure 1).

### 3.2. Differential Expression of AACs in Kidney Cell Lines in Response to Serum Deprivation

For evaluating how the expression levels of AACs change in non-cancer kidney cells (HK2) and cancer (CaKi-1 and RCC-Shaw) cells, we performed quantitative real-time PCR (qRT-PCR) analyses and western-blot (WB) analyses of AACs expressed in the kidney in normal (complete medium) growth conditions and in starved (serum-free) growth conditions.

**Figure 2:**
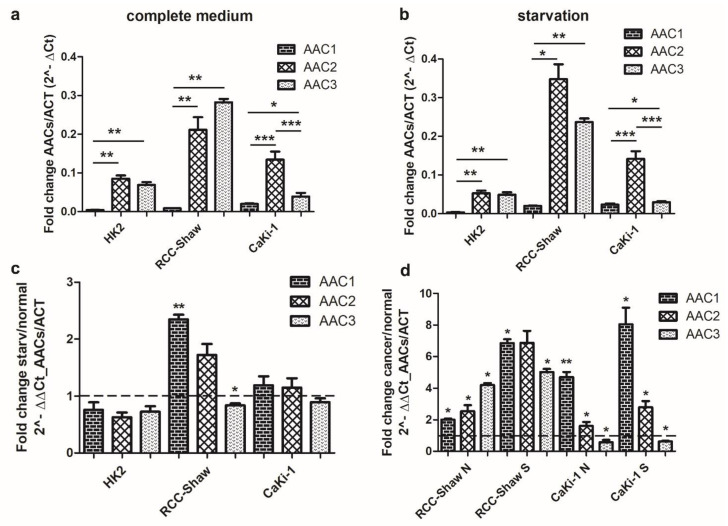
qRT-PCR of AAC1, AAC2, and AAC3 in non-cancer kidney cells (HK2) and cancer (CaKi-1 and RCC-Shaw) cells. Comparison of the AAC1, AAC2, and AAC3 gene expression levels between cells grown in complete medium (**a**), cells grown in serum-deprived medium (**b**), cells grown under starved (serum-free) conditions vs. normal (complete medium) conditions (**c**), and cancer cells vs. non-cancer cells are reported (**d**). Panel d: “N” indicates normal (complete medium) growth conditions, “S” indicates starved (serum-free) conditions. Data are presented as mean + SE of at least three independent experiments. The reported AAC expression levels were normalized to the nuclear housekeeping ACT gene. * *p* < 0.05; ** *p* < 0.01, *** *p* < 0.001, nonparametric Wilcoxon two-tailed test between starved and physiological conditions. “Starvation” indicates cells grown in a serum-free medium. “ACT” indicates actin.

It was observed that AAC1 is much less expressed than AAC2 and AAC3 in the three investigated cell lines, both in normal growth conditions (Figure 2a) and in serum-free medium (Figure 2b).

It was also observed that AAC1, AAC2, and AAC3 are similarly, or slightly less, expressed in HK2 cells, when HK2 cells are grown in serum-free medium, compared to HK2 grown in complete medium (Figure 2a–c).

In CaKi-1 cells grown in serum-free medium, AAC1-2 are slightly more expressed than their counterparts in the same cells grown in complete medium (Figure 2c), whereas AAC1 and AAC2 in RCC-Shaw cells, grown in serum-free medium, are significantly more expressed than their counterparts in the same cells, grown in complete medium. RCC-Shaw cells grown in serum-free medium, showed a low decrease of AAC3 expression levels (Figure 2c), at variance with RCC-Shaw cells grown in complete medium.

Serum-starvation caused a significant increase in the expression levels of AAC1-3 in RCC-Shaw cells, or AAC1-2 in CaKi-1 cells, at variance with their counterparts in non-cancer HK2 cell line (Figure 2d). AAC3 appears to be less expressed in CaKi-1 cells in the serum-free medium at variance with their counterpart in non-cancer HK2 cells (Figure 2d).

Our results point to serum-free medium as the major inducer of AAC1 in the investigated RCC-Shaw and CaKi-1 cancer cells, although AAC1 is the paralog less expressed in all the investigated cell lines.

### 3.3. AAC Protein Expression in Non-Cancer and Cancer Kidney Cells

**Figure 3:**
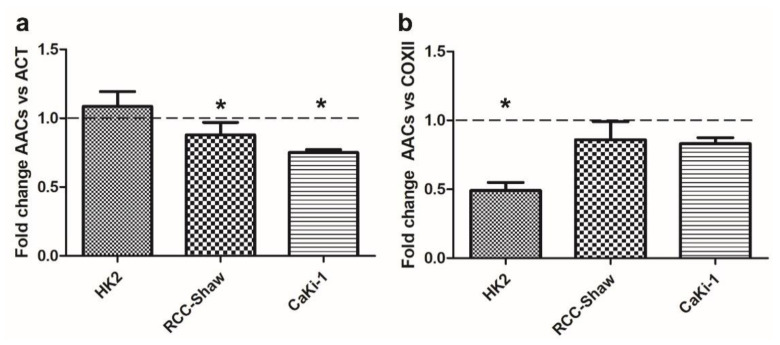
Ratio of AACs protein content estimated through WB in HK2, RCC-Shaw, and CaKi-1 cells grown in serum-free medium versus complete medium conditions with respect to actin content (panel **a**) or COXII content (panel **b**). Data are presented as mean + SE of at least three independent experiments. * *p* < 0.05; nonparametric Wilcoxon two-tailed test between starved and physiological conditions. For a representative WB see Supplementary Figure S3. The protein concentration from extraction assays was reported in Supplementary Table S2.

WB analyses confirmed that AACs protein content is significantly reduced in cancer (RCC-Shaw and CaKi-1) compared to non-cancer cell lines (HK2). Notably, HK2 cell lines do not display any significant variation in AAC levels in answer to serum deprivation (Figure 3a).

Conversely, WB reveals that AACs protein content normalized to the mitochondrial COXII content is significantly reduced in non-cancer cells at variance with what was observed in the investigated cancer cell lines (RCC-Shaw and CaKi-1) that do not show any significant variation following serum deprivation (Figure 3b).

### 3.4. Survival Rate

**Figure 4:**
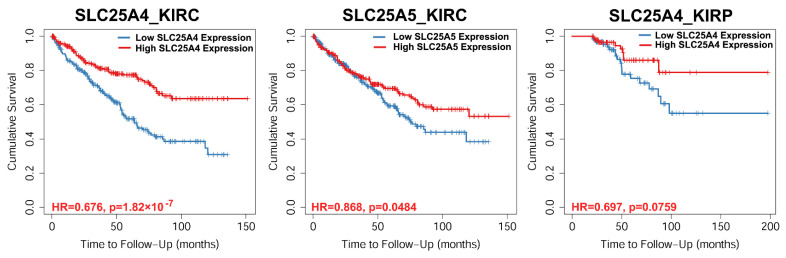
Survival analysis of TCGA kidney cancer patients as a function of the expression of the indicated AACs. Cumulative survival probability was evaluated in patients with the highest and lowest 50% gene expression using the Kaplan-Meier curve and Cox proportional hazard model as calculated by Timer2. Hazard ratio (HR) and the significance of the outcome (p) are also indicated.

Survival analyses conducted on TCGA datasets of kidney cancer, that is, 533 KIRC samples and 290 KIRP samples, showed that low expression levels of *SLC25A4_AAC1* and *SLC25A5_AAC2* correspond to low cumulative survival probability of KIRC (see *SLC25A4* and *SLC25A5* panels of Figure 4) and KIRP (*SLC25A4* panel of Figure 4) patients.

It appears that also the low expression of *SLC25A6_AAC3* in KIRC patients and *SLC25A5_AAC2* in KIRP patients corresponds to a slightly lower cumulative survival probability of the corresponding affected patients (Supplementary Figure S2).

### 3.5. In Vitro Caspase-9 Activation in the Investigated Cell Lines

**Figure 5:**
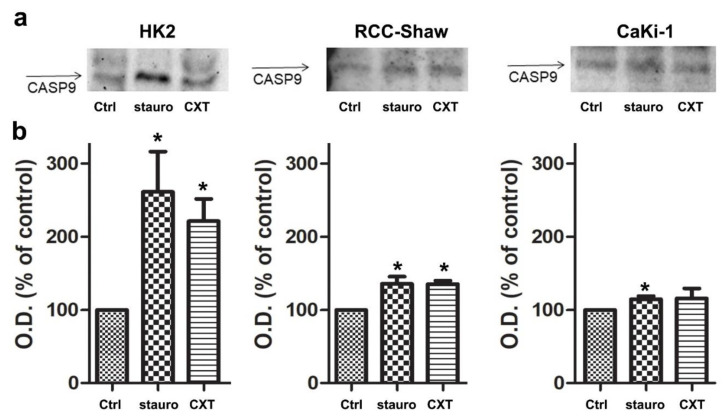
Quantification of Cleaved-CASP9 in HK2, RCC-Shaw, and CaKi-1 cells. Panel (**a**). Representative western blots are reported. Panel (**b**). Data obtained by the optical density of cleaved-CASP9 immunoreactivity bands were expressed as a percentage of the controls. Values represent mean ± S.E. (*n* = 3). * *p* < 0.05, nonparametric Wilcoxon test between cell lines treated with staurosporine (stauro) or carboxyatractyloside (CXT) and non-treated cells (Ctrl).

Then we tested the ability of AAC inhibitors to induce apoptosis in the evaluated cell lines by WB monitoring of Caspase-9 [38] as an indicator of intrinsic apoptotic (mitochondrial) pathway activation. 30μg of each sample underwent electrophoretic separation using polyacrylamide gel followed by membrane transfer. The antibody recognizes the Aspartate present in the protein fragment yet fails to bind in an absolute way to the only subunit of Caspase-9 with a molecular weight of 37 kDa, but also that of 35 kDa, highlighting in many cases, a double banding in plate analysis. Incubation with staurosporine (2 µM, 3 h incubation) or CXT (40 µM, 24 h incubation) led to mitochondrial apoptosis activation through the Caspase-9 cascade in all the investigated cell lines (Figure 5). Notably, the investigated kidney cancer-cells appeared to be less sensitive to the mitochondrial (intrinsic) apoptosis initiator “staurosporine” and to the AAC highly selective inhibitor CXT (Figure 5), compared to non cancer cells.

## 4. Discussion

Most current cancer chemotherapeutics cause cell death via apoptosis through two main apoptotic pathways: the extrinsic or death receptor pathway, and the intrinsic or mitochondrial pathway [1,2]. It is known that the mitochondrial apoptotic pathway is triggered by the opening of a large protein complex known as mPTP, although there is still much debate about mPTP components [5,39,40,41,42]. The human AACs [3,4] are among the most studied mPTP components/effectors and play a key role in regulating mPTP opening and thus in triggering the mitochondrial apoptotic pathway [5,6,7,39,40,41,43].

AACs play a crucial role in cell viability because they export ATP from the mitochondrial matrix to the cytosol and import ADP from cytosol to mitochondrial matrix [3,4] and have been largely investigated as a crucial component or effector of mPTP opening/regulation [5,44].

The irreversible inactivation of AACs caused by CXT, accidentally ingested by healthy individuals, was identified as the primary cause of poisoning at the physiological level and mitochondrial dysfunction at the molecular level [18,19,21,22,24,45]. The concept of AACs as a therapeutic target for anti-cancer therapy has been validated in a preclinical mouse model of tumor xenograft [21,45].

Given the crucial role played by AACs in energy production and mPTP opening/regulation [5,6,7,39,40,41,43,46] and the proposed pro/anti-apoptotic features of AACs [47], we screened the TCGA database for estimating/updating what is known about the alteration of the expression levels of AACs in cancer tissues by using Timer2 [29], for evaluating if AACs might represent a new molecular target for treating cancer.

The performed screening allowed verifying that AACs are differentially expressed in several cancer tissues compared to their normal counterparts. Indeed, breast cancer (BRCA), colon adenocarcinoma (COAD), head-neck squamous carcinoma (HNSC), kidney chromophobe renal cell carcinoma (KICH), kidney renal clear cell carcinoma (KIRC), kidney renal papillary cell carcinoma (KIRP), lung squamous cell carcinoma (LUSC), prostate adenocarcinoma (PRAD), and stomach adenocarcinoma (STAD) “tumor vs. normal” tissues pairs show at least two AACs significantly differentially expressed.

Given that among the available tissues, kidney “cancer/normal” tissue pairs showed a significant differential expression of at least two AACs in all the available tissue samples, we decided to estimate AACs expression in kidney “cancer/non-cancer” cells. Furthermore, we estimated how serum deprivation, known to induce anaerobic metabolism in a mimic of the Warburg effect [28,48,49,50,51,52,53,54] might influence AAC expression in non-cancer and cancer kidney cell lines.

With this aim, we chose to estimate AAC expression levels in a non-cancer kidney cell line (HK2) and in two kidney cancer cell lines (CaKi-1 and RCC-Shaw) grown in complete medium or in serum-free medium.

Our show that AAC2 and AAC3 are significantly more expressed than AAC1 in each investigated cell line grown in complete medium. Serum starvation causes a weak decrease in the three AAC transcripts in the non-cancer HK2 cells, whereas a significant increase of AAC1 and a weak decrease in AAC3 is observed in the RCC-Shaw cell line. On the other hand, CaKi-1 cells show a weak (not significant) increase in the expression of AAC1 and AAC2, whereas the AAC3 transcript appears unchanged.

Notably, comparing the expression levels of the investigated AACs in cancer versus non-cancer cell lines, it is observed that the three AACs are significantly more expressed in RCC-Shaw cells with respect to HK2 cells. Also, CaKi-1 cancer cells show AAC1 and AAC2 upregulated, whereas AAC3 appears weakly downregulated compared to non-cancer HK2 cells.

In addition, serum starvation causes a further significant increase of two AAC expression levels in RCC-Shaw cells (AAC1 and AAC3) and CaKi-1 cells (AAC1 and AAC2) with respect to non-cancer HK2 cells grown in the same conditions.

Following AAC transcript evaluation, we proceeded with the estimation of the total AAC protein content. It was necessary to estimate the total AAC protein content because the full-length sequences of three AAC proteins share more than 94% of identical amino acids, and unfortunately, no specific AAC antibody exists that is able to discriminate between the three investigated paralogs. Thus, WB analyses revealed that the total AAC protein content normalized for (the nucleus encoded) ACT within HK2 cells does not change in serum-starved growth conditions, whereas AAC protein content results significantly reduced in RCC-Shaw and CaKi-1 cells. When the normalization is performed with respect to the (the mitochondrial encoded) COXII subunit content, it is shown that starvation causes a reduction of the total AAC protein content in HK2 cells, at variance with RCC-Shaw and CaKi-1 cell lines, which do not show any significant difference in the total AAC protein content normalized for the COXII content.

The different observed trends in the total AAC protein content might be ascribed to the different pathways regulated by serum deprivation. Indeed, it is expected that proteins coded by nuclear genes are not reduced as a consequence of the starvation in the investigated non-cancer cells, whereas it appears that the total AAC protein content, normalized for ACT is reduced by serum-starvation in cancer cells.

Conversely, when the total AAC protein content is normalized for the mitochondrial COXII reference protein, it is observed that AAC protein content is significantly reduced in HK2, despite what was observed for the weakly reduced AAC protein content, as compared to COXII, as a consequence of the starvation in the investigated cancer cells.

It might be speculated that serum-starvation can be implicated in the increase of the mitochondrial COXII subunit content as a consequence of the stimulation of mitochondrial biogenesis [55] in HK2 non-cancer cells, at variance with kidney cancer cell lines, known to be glycolytic cancers with impaired mitochondria [56,57,58]. The increased mitochondrial biogenesis would result in an increased COXII protein content that would be coherent with the decreased total AAC protein content (normalized with respect to COXII) observed in HK2 cells grown in serum-free medium.

It should be noticed that the total AAC protein content decreases in the investigated kidney cancer cell lines (when normalized for ACT), according to what is expected from glycolytic cancers with impaired mitochondrial function [56,57,58].

**Figure 6:**
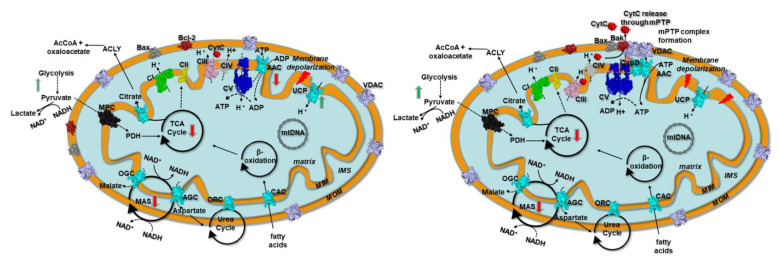
Scheme of mitochondria with a set of representative proteins, pathways, and cycles in kidney cancer prior and after mPTP formation. Left panel. Kidney cancer mitochondria resistant to mitochondrial apoptosis. Right panel. Kidney cancer mitochondria after mPTP formation. Respiratory chain complexes, mitochondrial transporters, and other proteins are reported in surf representation and labeled. ATP synthase (CV) is reported in blue. Mitochondrial carriers are reported in cyan. VDAC is reported in pink. Bax and Bak/Bcl-2 are reported in dark-grey and firebrick, respectively. MPC is reported in black. Complex I (CI), complex II (CII), complex III (CIII), and complex IV (CIV) are reported in green, yellow, magenta, and grey, respectively. Black circular arrows indicate cyclic pathways. Red arrows indicate impaired pathways or reactions. Green arrows indicate upregulated pathways or reactions. Black dashed lines indicate impaired reactions. Abbreviations: MIM: mitochondrial inner membrane; MOM, mitochondrial outer membrane; IMS, intermembrane space; AAC, ADP/ATP carrier; CAC, carnitine/acyl-carnitine carrier; ORC, ornithine carrier; AGC, aspartate/glutamate carrier; OGC, malate/2-oxoglutarate carrier; MAS, malate/aspartate shuttle; TCA, tricarboxylic acid cycle; Bax, Bcl-2 associated X protein;Bak, Bcl-2 antagonist/killer-1; Bcl-2, B-cell lymphoma-2; MPC, mitochondrial pyruvate carrier; UCP, uncoupling protein; CypD, cyclophilin D; CytC, cytochrome C; VDAC, voltage-dependent anion channel; AIF, apoptosis-inducing factor; mPTP, mitochondrial permeability transition pore.

Indeed it is expected that the investigated kidney cancer cell lines, as reported for other related renal cancers [26,27,56,57,58,59,60], behave as glycolytic cancers, producing a high amount of lactic acid, which suggests a lowered mitochondrial function, coherently with a Warburg cancer [61]. Glycolytic cancers show frequent mitochondrial impairment, a strong reduction in the transfer of reducing equivalents through the malate/aspartate shuttle (MAS), and the increase of lactic acid production [62,63,64,65,66] (Figure 6).

However, other alterations have been well described in tumor cells/tissues, beyond the Warburg effect [61,67]. Indeed, it is known that several tumors produce ATP through oxidative pathways along with the upregulation of lipid biogenesis pathways, citrate export, and the involvement of citrate lyase and isocitrate dehydrogenases (Figure 6), observed dysregulated/mutated in several cancers [66,68,69,70,71,72,73,74,75].

Different pathway alterations might be responsible for the differential expression of AACs observed in our screening of the TCGA database. The differential expression of AACs in the different cited cancer/non-cancer tissue pairs should reflect specific metabolic adaptations in response to specific cancer driver mutations or gene expression alterations, as already observed in cell-based assays [25]. AAC1 and AAC3 were proposed as pro-apoptotic proteins, whereas AAC2 and AAC4 were proposed as anti-apoptotic oncoproteins [47]. More in general, it is expected that glycolytic cancers, producing ATP mainly from glycolysis and showing impaired mitochondria [62,63,76,77], will have a higher likelihood to show downregulated AACs, whereas other oxidative cancers, producing ATP mainly from fatty acid oxidation [78,79,80], by increasing mitochondrial performances, might show upregulated AACs, but more dedicated analyses are necessary for clarifying the role of each AAC coding gene in the different cited cancer types.

Notably, the cumulative survival probability of patients affected by the investigated kidney cancers as a function of AAC expression levels, shows that the survival rate decreases with the decrease of AAC1 expression both in KIRC and KIRP affected patients and with the decrease of AAC2 expression in KIRC affected patients.

In this context, it appears that the decrease of AAC1 expression (and AAC2 at a lower extent) might be used as a genetic marker, among other already context-specific characterized genes [81], for estimating/predicting the survival of patients affected by renal cancer or the aggressiveness of the investigated kidney cancers.

In light of the above-reported observations, the employment of selective AAC inhibitors (such as CXT and its derivatives) [4] entrapped in *ad-hoc* mitochondrial delivery systems [82], in combination with traditional therapies, might represent a pharmacological option as it can selectively kill cancer cells that cannot be re-programmed.

Thus, we showed that mitochondrial apoptosis might be triggered with relatively low amounts of CXT (40 µM), also in those cell lines expressing low AAC levels, as compared to staurosporine (2 µM). It would be expected that CXT, showing a Ki for AACs equal to 4 nM [4], would trigger mitochondrial apoptosis at lower concentrations in the investigated kidney cell lines.

On this concern, it was proposed that the resistance to CXT mediated mitochondrial apoptosis in cancer cells might be ascribed to the removal of CXT from tumor cells via multidrug-resistant ATP-binding cassette (ABC) transporters [83] or to an alternative mechanism of mitochondrial ADP/ATP transport in tumor cells, not targeted by CXT [84]. However, we retain that the higher concentration of CXT, as compared to staurosporine, requested for triggering mitochondrial apoptosis can be ascribed to the poor permeability of this compound through the plasma membrane of the intact cells [85].

Conversely, permeability problems and, at the same time, the indiscriminate toxicity of AAC inhibitors [4,23], able to target simultaneously AACs from several tissues, might be avoided by chemical conjugation of the CXT (and future CXT structurally related ligands) to a specific monoclonal antibody (mAb) directed against plasma membrane receptors expressed selectively on cancer cells [86,87,88]. The mAbs should grant the specific delivery of the investigated inhibitors to cancer cells expressing specific (or highly selective) receptors on the tumor cell surface. Conjugation to mAbs would favor plasma membrane permeation and would allow using the minimal cytotoxic concentration of the inhibitor conjugated to the mAb, for reducing the alteration of the mAb pharmacokinetics in vivo.

Reducing the concentration of the inhibitor conjugated to the mAb would also allow to not alter mAb binding affinity for the receptor on the target cell, which would be expected in case of a large number of drug molecules conjugated to the mAb [89].

In conclusion, it is expected that the synergic employment of AAC directed inhibitors conjugated to highly cancer-selective mAbs, combined with traditional therapies, might be more effective than other chemotherapy approaches because it would be targeted chemotherapy that directly triggers mitochondrial apoptosis by targeting one of the main modulators of mPTP opening.

## Data Availability

Not applicable.

## Supplementary Materials

The following are available online at https://www.mdpi.com/2218-273X/11/1/38/s1, Supplementary Figure S1: SLC25A31_AAC4 expression levels in 21 “cancer vs. normal” tissue pairs available on TCGA, analyzed through Timer2. Supplementary Figure S2: Survival difference related to AACs expression in the investigated cancer tissues, Supplementary Figure S3: AACs protein content estimated through WB in HK2, RCC-Shaw and CaKi-1 cells grown in complete medium or starvation (serum-free medium, starved cell lines are those indicated with “star” in the label) with respect to actin or COXII content. M = marker; MW Molecular Weight marker; PageRuler™ Prestained Protein Ladder; Thermo Fisher Scientific). Supplementary Table S1: Pairs of primers used for qRT-PCR Supplementary Table S2: RNA and proteins extracted from the investigated cell lines (from a representative replicate) for qRT-PCR and WB analyses.
