# Supplementary material for: Differential Expression of ADP/ATP Carriers as a Biomarker of Metabolic Remodeling and Survival in Kidney Cancers
Source: Biomolecules. 2020 Dec 30;11(1):38. doi: 10.3390/biom11010038 (PMC7824283; doi:10.3390/biom11010038)
Supplement: Supplementary file 1 [file biomolecules-11-00038-s001.pdf]

Supplementary Materials

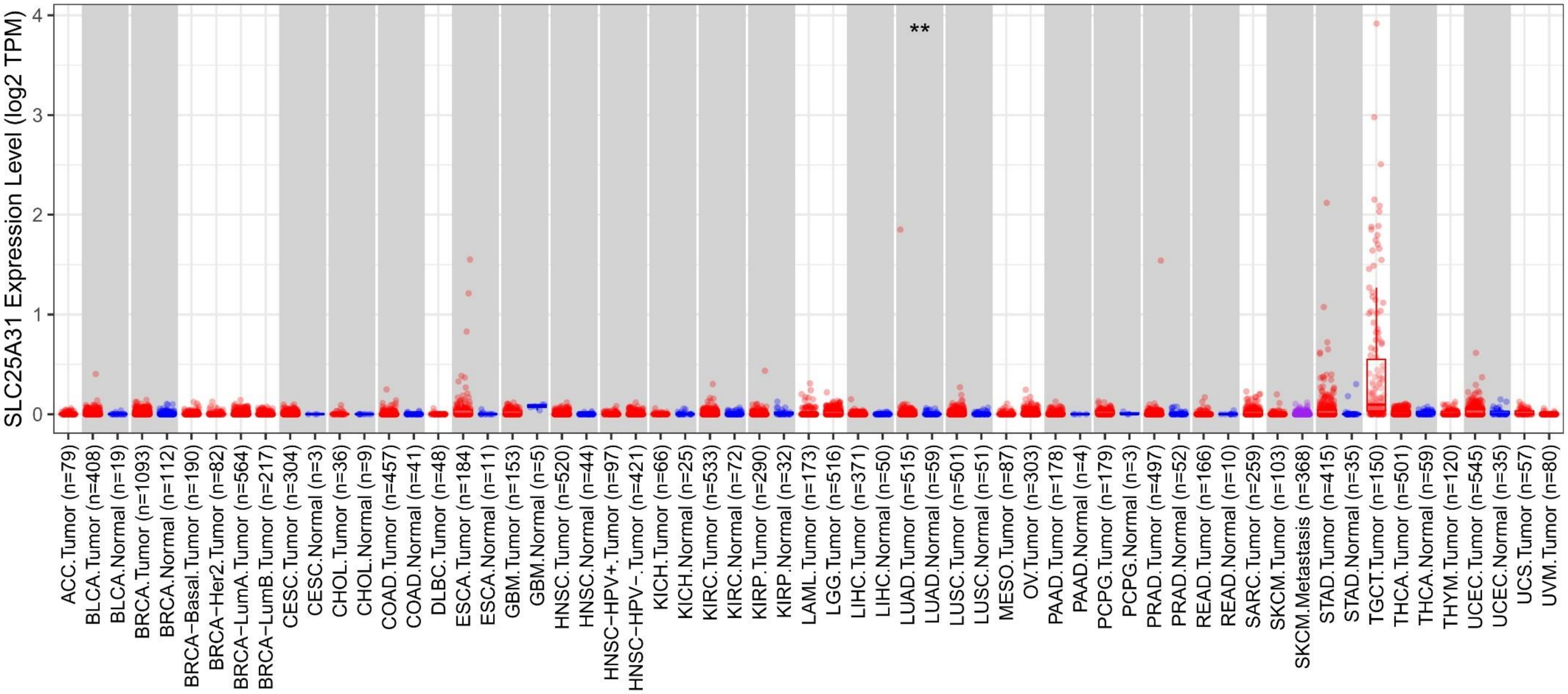

Supplementary Figure S1. SLC25A31_AAC4 expression levels in 21 “cancer vs healthy” tissue pairs available on TCGA, analysed through Timer2.

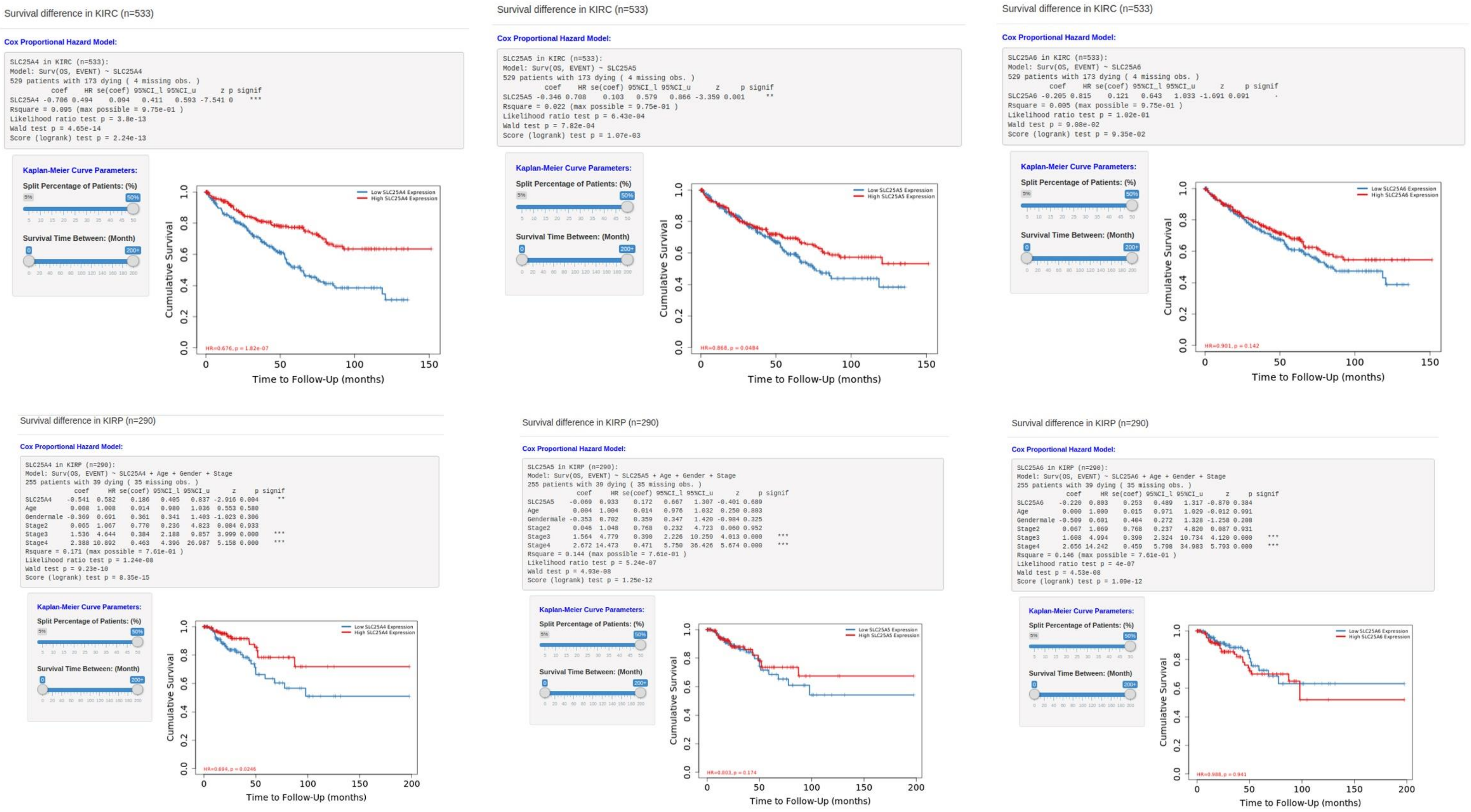

Supplementary Figure S2. Survival difference related to AACs expression in the investigated cancer tissues

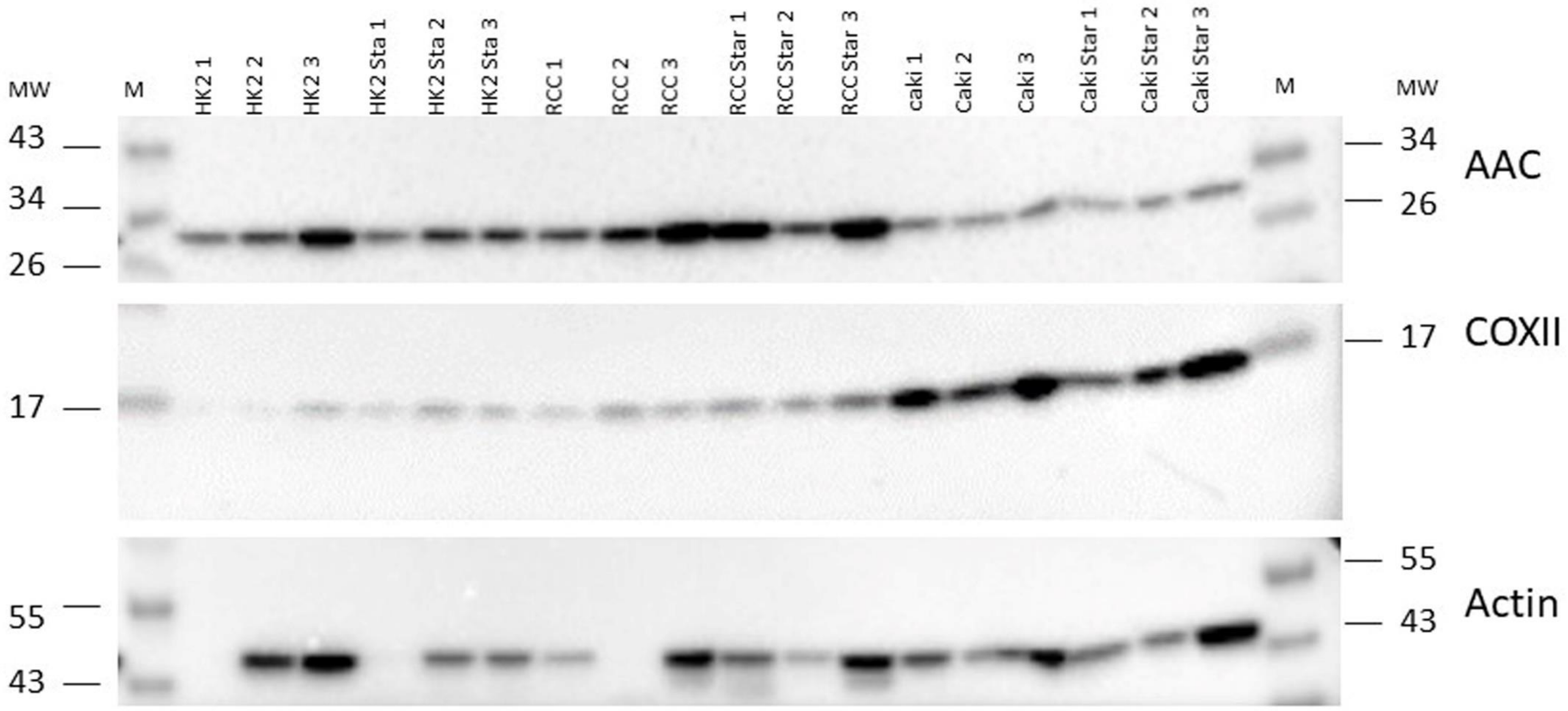

Supplementary Figure S3. AACs protein content estimated through WB in HK2, RCC shaw and CaKi-1 cells grown in complete medium or starvation (serum-free medium, starved cell-lines are thos indicated with "star" in the label) with respect to actin or COXII content. M = marker; MW Molecular Weight marker; PageRuler™ Prestained Protein Ladder; Thermo Fisher Scientific).

Supplementary Table S1. Pairs of primers used for qRT-PCR

**Name**	**Sequence**	**Length**
ACT-FOR	5′- GAAGATCAAGATCATTGCTCCT -3′	111
ACT-REV	5′- TACTCCTGCTTGCTGATCCA -3′	
AAC1-FOR	5′- CTGGTGTCCTACCCCTTTGA-3′	129
AAC1-REV	5′- CTTGGCTCCTTCGTCTTTTG -3′	
AAC2-FOR	5′- ATCTACCGAGCCGCCTACTT -3′	105
AAC2-REV	5′- GACAGTCTGTGCGATCATCC-3′	
AAC3-FOR	5′- TGCAGGGCATCATCATCTAC-3′	106
AAC3-REV	5′- ATCATCCAGCTCACCACGAT -3′	
AAC4-FOR	5′- AAACCTGGCTTCTGGTGGAG -3′	118
AAC4-REV	5′- GAATTGTCGCTCCTCAGGAC-3′	

Supplementary Table S2. Table showing RNA and proteins extracted from the investigated cell-lines (from a representative replicate) for qRT-PCR and WB analyses.

Cell-line >>>>>>>>>	HK2 complete medium	HK2 serum-free medium	RCC-shaw complete medium	RCC-shaw serum-free medium	CaKi-1 complete medium	CaKi-1 serum-free medium
**Number of cells for each T75**	3,8 x 10^6	4,8 x 10^6	11,8 x 10^6	12 x 10^6	12 x 10^6	12 x 10^6
**Number of cells for RNA extraction**	1 x 10^6	2 x 10^6	2 x 10^6	2 x 10^6	2 x 10^6	1 x 10^6
**Eluted volume (uL)**	40	80	80	80	80	80
**Abs (260 nm)**	2,03	0,952	0,314	0,667	0,453	0,341
**260/280**	2,09	1,94	1,72	1,96	1,92	1,97
**Conc NanoDrop (ng/uL)**	81,1	38,1	23,3	1,03	43	31
**Dilution sample**	10	10	10	10	10	10
**Conc RNA (ng/uL)**	811	381	233	275	430	316
**Number of cells for WB**	2,8 x 10^6	2,8 x 10^6	9,8 x 10^6	10 x 10^6	10 x 10^6	10 x 10^6
**Volume Ripa Buffer (uL)**	150	100	300	300	400	400
**Sonication time (sec)**	3	6	3	6	5	5
**Abs (595 nm)**	0,72	0,36	0,502	0,179	0,651	0,393
**cuvette concentration (mg/mL)**	0,716	0,678	0,985	0,237	0,65	0,415
**Dilution sample**	10	10	10	10	10	10
**Protein concentration (mg/mL)**	7,16	6,78	9,85	2,73	6,5	4,2
